# Extended liquid state machines for speech recognition

**DOI:** 10.3389/fnins.2022.1023470

**Published:** 2022-10-28

**Authors:** Lucas Deckers, Ing Jyh Tsang, Werner Van Leekwijck, Steven Latré

**Affiliations:** imec IDLab, Department of Computer Science, University of Antwerp, Antwerp, Belgium

**Keywords:** spiking neural networks, liquid state machine, reservoir computing, sound processing, E/I balance, spike-frequency adaptation, neuronal diversity

## Abstract

A liquid state machine (LSM) is a biologically plausible model of a cortical microcircuit. It exists of a random, sparse reservoir of recurrently connected spiking neurons with fixed synapses and a trainable readout layer. The LSM exhibits low training complexity and enables backpropagation-free learning in a powerful, yet simple computing paradigm. In this work, the liquid state machine is enhanced by a set of bio-inspired extensions to create the extended liquid state machine (ELSM), which is evaluated on a set of speech data sets. Firstly, we ensure excitatory/inhibitory (E/I) balance to enable the LSM to operate in edge-of-chaos regime. Secondly, spike-frequency adaptation (SFA) is introduced in the LSM to improve the memory capabilities. Lastly, neuronal heterogeneity, by means of a differentiation in time constants, is introduced to extract a richer dynamical LSM response. By including E/I balance, SFA, and neuronal heterogeneity, we show that the ELSM consistently improves upon the LSM while retaining the benefits of the straightforward LSM structure and training procedure. The proposed extensions led up to an 5.2% increase in accuracy while decreasing the number of spikes in the ELSM up to 20.2% on benchmark speech data sets. On some benchmarks, the ELSM can even attain similar performances as the current state-of-the-art in spiking neural networks. Furthermore, we illustrate that the ELSM input-liquid and recurrent synaptic weights can be reduced to 4-bit resolution without any significant loss in classification performance. We thus show that the ELSM is a powerful, biologically plausible and hardware-friendly spiking neural network model that can attain near state-of-the-art accuracy on speech recognition benchmarks for spiking neural networks.

## Introduction

The last decade has witnessed the advent of power-efficient, neuromorphic hardware (Davies et al., 2018; Tang et al., 2019) and phenomenal advances in the accompanying software. Specifically, spiking neural networks (SNN; Gruning and Bohte, 2014) have led to great successes, matching the performance of deep neural networks on a selected set of classification benchmarks, at only a fraction of their computational demands (Yin et al., 2021). These results encourage research in neuro-inspired computing systems, especially given the growing demand for low-power inference at the edge (Cao et al., 2020). Backpropagation-through-time (BPTT) with surrogate gradients (Neftci et al., 2019) is the most widely used technique for training SNNs. The surrogate gradients provide a solution to overcome the non-differentiable threshold mechanism in spiking neurons, and allow gradients to propagate back through the network to update the weights. Recently, enhanced BPTT training methods have been introduced, such as modified loss functions for improved, online learning in SNNs (Yang et al., 2022b), robust, continual, meta-learning (Yang et al., 2022c), and backpropagation, based on temporal coding (Kheradpisheh et al., 2022).

Another popular and straightforward approach that enables learning with SNNs relies on reservoir computing, more precisely a liquid state machine (LSM; Maass et al., 2002). An LSM was originally presented as a biologically plausible model for a cortical microcircuit as it consists of a random, sparsely connected, recurrent, high-dimensional spiking reservoir with fixed synapses and a trainable readout layer. Considering the low training complexity and speed, as well as their compatibility with deployment on efficient neuromorphic hardware (Li et al., 2020; Wang et al., 2022), LSMs have become an attractive SNN model for low-power edge computing. Furthermore, despite their simple setup, LSMs have been established as a powerful spatio-temporal feature extractor with remarkable results on various tasks (Al Zoubi et al., 2018; Soures and Kudithipudi, 2019) even surpassing the performance of a large CNN+LSTM model on a radar-based gesture recognition benchmark (Tsang et al., 2021).

Recently, several approaches to improving the performance of LSMs have been proposed. Task-agnostic, data-driven training of the recurrent liquid weights (Jin and Li, 2016; Ivanov and Michmizos, 2021), continuous neuronal adaptation based on intrinsic neuronal plasticity (Zhang and Li, 2019a), reservoir autoregulation (Balafrej et al., 2022), liquid ensembles (Wijesinghe et al., 2019), and evolutionary optimization (Zhou et al., 2020) have all been shown to improve the basic LSM design, keeping its sparse properties. However, these enhancements come at the cost of increased (training) complexity and data-dependent tuning of the LSM parameters, eliminating some of its inherent advantages. None of these approaches have, however, reached state-of-the-art performances on benchmark data sets in comparison with SNNs trained with BPTT.

In this work, we propose the extended LSM (ELSM). All presented extensions to the base LSM are inspired by features commonly found in biological neurons and also known to improve feature encoding in biology. The first extension covers E/I (excitatory/inhibitory) balance. Neuroscientists have uncovered that the neural coding efficiency is maximized when excitatory and inhibitory presynaptic currents are balanced (Zhou and Yu, 2018). The resulting E/I balance is related to an optimized dynamical network chaos (Van Vreeswijk and Sompolinsky, 1996), called the edge of chaos. In previous research (Ivanov and Michmizos, 2021; Balafrej et al., 2022), edge-of-chaos dynamics were shown to improve the neural coding in an LSM in comparison with a randomly initiated liquids. The balance of excitatory and inhibitory currents can thus be seen as an often neglected heuristic for optimized coding in LSMs. Following on from the E/I balance, the second extension is to update the neuronal model by adding spike-frequency adaptation (SFA). This is a passive neuronal mechanism that updates the spiking threshold based on its previous spiking behavior. In previous research (Salaj et al., 2021), SFA was shown to improve the memory capabilities in an SNN, trained end-to-end with e-prop (Bellec et al., 2020). The third and final extension is to include neuronal heterogeneity (Perez-Nieves et al., 2021). This concept was shown to enhance the computational requirements and robustness in SNN classification tasks. The effect of these brain-inspired modifications on an LSM have currently not been addressed in research. In this work, generic benchmark speech recognition applications were selected for evaluation of the base LSM as well as the proposed extensions. These were selected since on-device computing is crucial for the privacy and security of sensitive speech data. The proposed extensions are however independent of the task at hand.

We show that each of our LSM extensions: achieving E/I balance, spike-frequency adaptation and neuronal heterogeneity, improve the general speech recognition capacity of an LSM, in terms of classification accuracy, throughout four data sets: Spiking Heidelberg digits (SHD), TI-46-Word, N-TIDIGITS, and Google Speech Commands (GSC). Furthermore, we illustrate that the optimal E/I balance can be found by regulating the random distributions of the input-liquid weights. The extensions presented in this work are shown to consistently improve the performance of the LSM and reach toward state-of-the-art accuracy, without the need of fine-tuned weights *via* BPTT. Lastly, we show that the resolution of the synaptic weights in the ELSM can be scaled to a 4-bit representation without significant loss in performance, making the ELSM a powerful and biologically plausible alternative to BPTT-trained networks.

## Materials and methods

In this section, we review the neuron and synapse models as well as the baseline LSM structure and training procedures used in this work. In addition, we discuss the proposed extensions, namely the addition of E/I balance, spike-frequency adaptation (SFA), and the diversification of the neuronal parameters in comparison with the baseline LSM model. To run our experiments, we used Python and the Brian2 neural simulator (Stimberg et al., 2019).

### Neuron and synapse model

In our LSM model, the neuronal dynamics are modeled based on conductance-based leaky integrate-and fire (LIF) neurons. In this standard model, similarly to the neuronal dynamics in our reference LSM (Maass et al., 2002), the membrane potential is described by (1).


(1) $$\begin{array}{l} \tau \frac{dV}{dt}=\left(E_{rest}-V\right)+g_{e}\left(E_{exc}-V\right)+g_{i}\left(E_{inh}-V\right) \end{array}$$

where *E*_*rest*_, *E*_*exc*_, and *E*_*inh*_ denote the resting membrane potential and the excitatory and inhibitory synapse equilibrium potentials, respectively. Other neuron parameters are *g*_*e*_(*g*_*i*_), the conductances of the excitatory (inhibitory) neurons and the membrane potential time constant, denoted by τ. If the membrane potential V crosses a fixed firing threshold *V*_*th*_, the neuron generates a spike. Then, the membrane potential is set to its reset potential *V*_*reset*_ and the neuron enters the refractory period, *t*_*ref*_, during which no further spikes can be emitted. The initial membrane potential is uniformly distributed as U[13.5, 15) mV and the neuronal conductances are initialized with zeros, as there were no previous spikes.

Synapse dynamics are modeled by changing conductances over time. When a presynaptic spike arrives, the neuron conductance is instantaneously increased by the synaptic strength, modeled by a fixed weight, *w*. Otherwise, the conductance exponentially decays over time, as denoted in (2).


(2) $$\begin{array}{l} \tau_{g_{e}}\frac{dg_{e}}{dt}=-g_{e} \end{array}$$

Here τ_*ge*_ indicates the time constant of the conductance of the excitatory neurons. Inhibitory neurons are modeled identically. The time constant τ_*ge*_ is typically longer for inhibitory neurons than for excitatory neurons. This is also supported by computational models that neuroscientists used to model real biological neuronal behavior (Destexhe et al., 2001; Brette and Gerstner, 2005). All neuron parameters were fixed over all our experiments and directly taken from a reference LSM implementation (Maass et al., 2002), apart from the conductance time constants, specified above. The neuron parameters used in this work are shown in Table 1.

**Table 1:** Conductance-based leaky integrate-and-fire neuron model: Parameter names and corresponding values.

**Name**	**Value**
Membrane rest potential, *E_*rest*_*	13.5 mV
Reset potential, *E_*reset*_*	13.5 mV
Excitatory rest potential, *E_*exc*_*	0 mV
Inhibitory rest potential, *E_*inh*_*	0 mV
Firing threshold, *V_*th*_*	15 mV
Membrane potential decay time constant, τ	30 ms
Excitatory decay time constant, *τg_*e*_*	3 ms
Inhibitory decay time constant, *τg_*i*_*	10 ms
Excitatory refactory period, *t_*ref, exc*_*	3 ms
Inhibitory refactory period, *t_*ref, inh*_*	2 ms

### Liquid state machine

The liquid state machine (LSM) is a biologically plausible model of a sparse, recurrent cortical microcircuit. An LSM consists of three main layers: the input, the liquid, and the readout/output layer. The input layer is sparsely connected in a random fashion to a reservoir of recurrently connected neurons, called the liquid. These input-liquid synapses are always excitatory and the probability of constructing the synaptic input-liquid connections, denoted by *p*_*in*_, is equal for the excitatory and inhibitory liquid neurons. The choice of the optimal *p*_*in*_ usually depends on the task at hand and is discussed in detail in the results section. The input-liquid synaptic weights are randomly sampled from a uniform distribution, U[0, 0.4], (U[0, 0.2]) for excitatory (inhibitory) synapses. The LSM parameters used in this work, were taken from our reference work (Maass et al., 2002), unless specifically stated otherwise. The synaptic delay for the input-liquid synapses, δ_*in*_, is equal to 1 ms.

**Figure 1:**
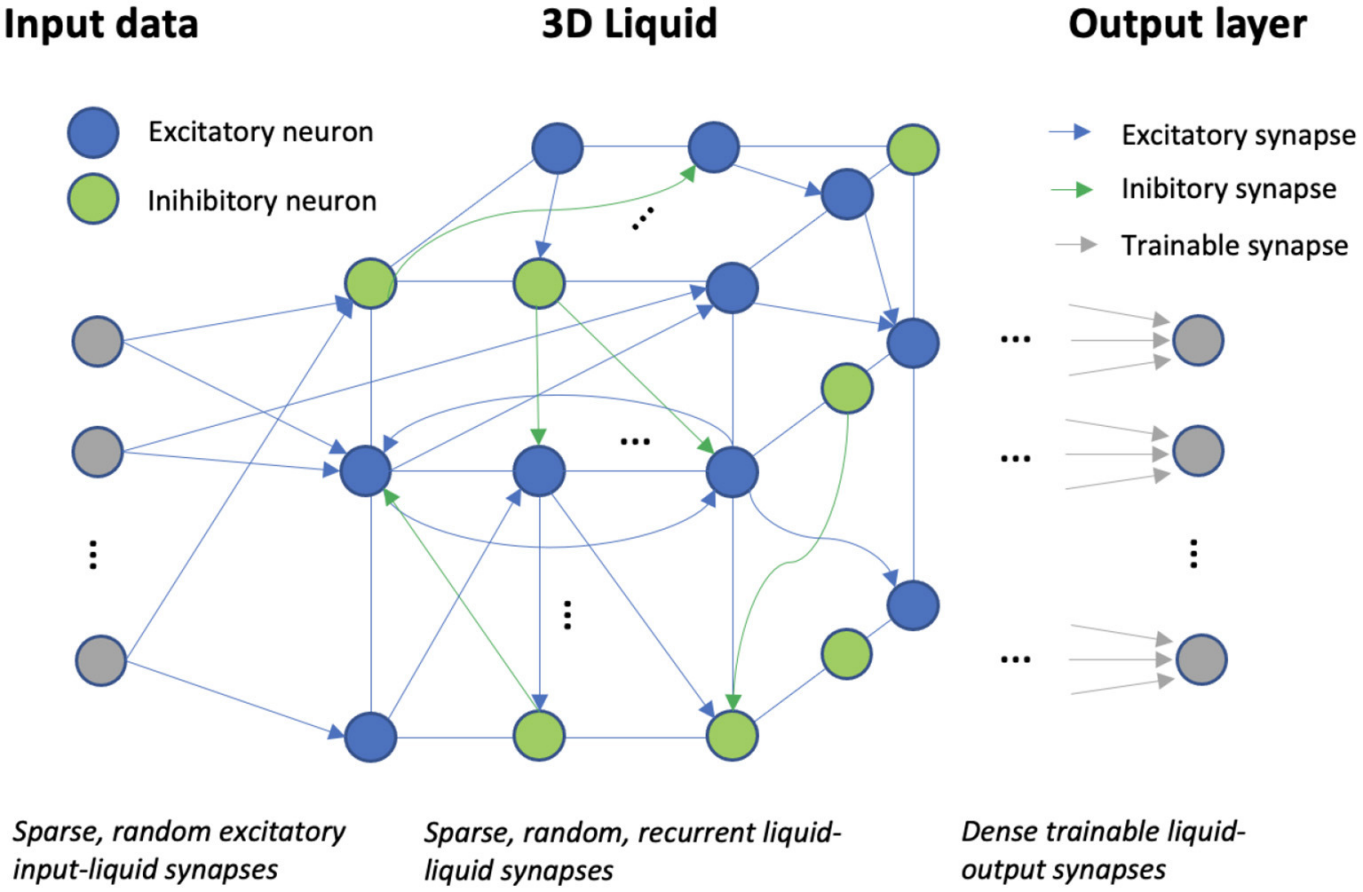
The liquid state machine: input data, spread across a number of channels, is projected *via* fixed, sparse, random synapses onto a sparse, high-dimensional, recurrent reservoir of spiking neurons. Training of the synaptic weights only happens in the dense output layer, modeled by a logistic regression.

The recurrent reservoir is modeled as a three-dimensional grid. Similarly to neural circuitry (Tsodyks et al., 2000) and as is common in LSM literature, the excitatory/inhibitory neuron ratio is set to 80/20%. The topology of the LSM is defined by *P*(*i,j*), the probability of a synaptic connection from neuron i to j, which depends on the Euclidean distance, *D*(*i,j*), between them:


(3) $$\begin{array}{l} P\left(i,j\right)=C*{e^{-\left(\frac{D_{\left(i,j\right)}}{\lambda }\right)}}^{2} \end{array}$$

where λ regulates the average number of synapses per neuron and the average distance between 2 neurons that are connected. The parameter C regulates the synaptic connection probability, which depends on the synapse type. The full parameter setup of the LSM is shown in Table 2. The synaptic delay, δ, in the reservoir is typically longer for EE (excitatory-excitatory) synapses than for the other types. The Euclidean distance-based design of the LSM topology, with a low λ naturally leads to a sparse reservoir, where every neuron is connected to another by ~9.5 recurrent synapses for a liquid that contains 2000 neurons. Similarly, the synaptic weights depend on the type of synapse. More precisely, the recurrent liquid-liquid weights are randomly sampled from a uniform distribution, depending on the type of synapse: U[0, 0.6] (EE), U[0, 0.4] (IE), U[0, 1.2] (IE), U[0, 0.4] (II). Note that spikes originating from inhibitory neurons decrease the membrane potential in the post-synaptic neuron. All synaptic weights are fixed after creation. An overview of the full LSM setup is shown in Figure 1.

**Table 2:** Liquid state machine model setup: Parameter names and corresponding values.

**Name**	**Value**
Global connectivity parameter, λ	2
Excitatory-excitatory parameter, *C*_*EE*_	0.3
Excitatory-inhibitory parameter, *C*_*EI*_	0.2
Inhibitory-excitatory parameter, *C*_*IE*_	0.4
Inhibitory-inhibitory parameter, *C*_*II*_	0.1
Input-liquid delay, δ_*in*_	1.0 ms
Excitatory-excitatory delay, δ_*EE*_	1.5 ms
Excitatory-inhibitory delay, δ_*EI*_	0.8 ms
Excitatory-inhibitory delay, δ_*IE*_	0.8 ms
Inhibitory-inhibitory delay, δ_*II*_	0.8 ms

The readout layer consists of a classifier, which is supervisedly trained. In general, the readout is memoryless and thus does not retain any memory of the previous states in the dynamical system. In this implementation, the readout is trained on the spike counts of the excitatory neurons for the duration of any sample. The resulting feature vector thus has size equal to the number of excitatory neurons and contains integer count values. A Logistic Regression classifier was selected because of its simplicity and to showcase the power of the non-linear separation carried out by the LSM.

### Bio-inspired liquid state machine extensions

In this section, we discuss three novel extensions to the baseline LSM model. These extensions are generic and not specifically tailored to one specific application. The methods in this work differ from current trends in liquid state machine research, where mainly the liquid is trained through local learning rules or optimized *via* evolutionary optimization methods. In these methods, the liquid is adapted to the task of interest at the cost of increased training time and complexity. Furthermore, in this work, all proposed modifications to the baseline liquid state machine are based on insights from neuroscience. The extended liquid state machine (ELSM) thus remains a biologically plausible model of cognition.

#### E/I balance

The first extension covers the neuronal excitatory/inhibitory (E/I) balance. In biological sensory processing, the interplay between synaptic excitatory and inhibitory stimulation is found to be well-balanced. This E/I balance, measured through the average ratio of excitatory to inhibitory synaptic conductances over time, plays a crucial role in efficient neural coding, information processing and network feature selectivity (Zhou and Yu, 2018). Moreover, in Kim and Sejnowski (2021), the authors show that strong inhibitory signaling is crucial for temporal processing in the cortex. In previous work (Van Vreeswijk and Sompolinsky, 1996), network balance, in form of the net synaptic input mean current and its fluctuations, was related to deterministic chaos. In more recent work (Ivanov and Michmizos, 2021), the authors describe E/I balance as a predictor for edge-of-chaos and therefore optimal liquid state machine dynamics.

In this work, we will focus on the effect of strong inhibitory signaling, in relation to E/I balance. We evaluate the influence of the input-liquid inhibitory synaptic weight strength on the E/I balance and consequently on the classification performance. By increasing the input current to the inhibitory liquid neurons, the total network activity becomes more sparse, leading to a more efficient coding scheme and a decrease in the number of spikes in the reservoir. Following results from the research works mentioned above, we presume that E/I balance is achieved at the point where the average net current over time approximates zero. At this point, the edge-of-chaos dynamics should lead to improved neural processing. The resulting classification performance, in comparison with results from the baseline LSM setup, is studied.

#### Spike-frequency adaptation

The second extension is called spike-frequency adaptation. Many of our daily tasks, such as speech recognition, include computing on features that are temporally distanced and thus require retaining memory in some way. These tasks are thus based on integration and processing on different timescales. Current spiking neuron models do not include any methods for such processing at a neuronal level for longer timescales, since spikes, and membrane potentials are updated at millisecond levels and leakage prevents any memory capabilities. From neuroscience (Gutkin and Zeldenrust, 2014), it is however known that both neurons and synapses exhibit slower dynamical processes, such as adaptation. These mechanisms can be related to processing at longer timescales. One of these processes is called spike-frequency adaptation (SFA), where the past firing activity of a neuron leads to an increase of the firing threshold *V*_*th*_. SFA can also be modeled by including an adaptation current. From a computational point of view, we chose to include the simpler adaptive threshold model for our experiments.


(4) $$\begin{array}{l} \tau \frac{dV}{dt}=\left(E_{rest}-V\right)+g_{e}\left(E_{exc}-V\right)+g_{i}\left(E_{inh}-V\right) \end{array}$$

(5) $$\begin{array}{l} V_{th}=V_{th,base}+V_{th,sfa} \end{array}$$

(6) $$\begin{array}{l} \tau_{sfa}\frac{V_{th,sfa}}{dt}=-V_{th,sfa} \end{array}$$

In biology, the SFA is known to enhance short-term memory (Marder et al., 1996; Turrigiano et al., 1996). Generally, the inclusion of SFA in the neuron model reduces the firing activity, which leads to an improved coding efficiency. Moreover, in previous works (Salaj et al., 2021; Shaban et al., 2021), SFA showed to improve the memory capacity of a recurrent spiking neural network, trained with e-prop, a biological approximation of the backpropagation algorithm. The authors have also shown that the classification results are optimized when the time constant of this decaying threshold, τ_*sfa*_, is equal to the expected duration when memory is required.

In this work, the LIF neuron model is adjusted to include SFA. As shown in Equation (4), the baseline spiking neuron model is updated by adding a variable threshold. Whenever an excitatory neuron fires, its firing threshold *V*_*th*_ is increased by a fixed value, which was set to 1. Otherwise, the threshold decays back to its original value, equal to the original *V*_*th,base*_, 15 *mV*. There is thus both a constant baseline and a variable, spike-dependent component. Since the expected duration is unknown for the features in our classification benchmark tasks—i.e., speech recognition—the time constant τ_*sfa*_, is uniformly set within the expected range where memory can be required. Similarly to biological neuronal networks, SFA is only applied to excitatory neurons.

#### Neuronal heterogeneity

The last extension is the inclusion of liquid heterogeneity—i.e., dissimilar neurons in the recurrent reservoir. As was previously shown (Maass et al., 2002), biological neural microcircuits behave like ideal liquids for computing with spatio-temporal features because of their large variety in neuron and synapse properties, as well as the variety of time constants, that characterize their interactions (Gupta et al., 2000). The diversity of computational elements improves the variety in the dynamical neuronal behavior and its subsequent feature extraction capabilities. In this work, instead of employing a range of different neuron or synapse models, which would lead to a more complex hardware implementation, we explore the heterogeneity of time constants. Similar to the work in Perez-Nieves et al. (2021), where heterogeneity is exploited in BPTT learning, we focus on the heterogeneity in τ, the membrane potential time constant. A shorter τ leads to a more responsive neuron, which means that the neuron will react stronger to incoming spikes, but it will also leak faster. This diversity will be compared to the original baseline, where τ is fixed.

### Experimental setup

#### Data sets used for validation

In order to show that the effects of the proposed LSM extensions are generic and robust, we selected four speech data sets for evaluation: (1) The Spiking Heidelberg Digits data set, a commonly used benchmark for SNN; (2) TI-Alpha, an LSM benchmark data set; (3) Google Speech Commands, a more demanding, noisy speech data set; (4) N-TIDIGITS, a spoken digits data set, recorded on neuromorphic hardware.

- The Spiking Heidelberg Digits (SHD; Cramer et al., 2020) is a SNN benchmark data set, where spikes are directly given as input data. The data consists of German and English spoken digits (0–9 for both languages), converted into spike trains based on a detailed cochlea model. As in Cramer et al. (2020), two speakers were held out for the test data set, and 5% of samples from 10 other speakers were also added into the test data set. The resulting training and test sets consist of 8,156 and 2,264 samples, respectively, as provided with the data set.
- TI 26-word “alphabet set,” a subset of the TI46-word corpus (Liberman et al., 1993). The “alphabet set” (TI-alpha) consists of utterances from 16 speakers each speaking 26 utterances consisting of the letters “a” through “z.” There are 4,142 and 6,628 instances in the training and test data sets, respectively, as provided in the speech corpus.
- The Google Speech Commands (GSC) data set (Warden, 2018), is a classical speech recognition data set. Version 2 has 105,829 utterances from 2,618 speakers in total. This speech corpus consists of 35 different commands. In literature, different splits are typically made: the full 35 words task and 12 words subset task, where a couple of commands are selected. We validated the ELSM on the full 35 words task for comparison with the state-of-the-art in spiking neural network models. Each utterance is 1 s long, and the sampling rate is 16 kHz. The data set is divided into a training, validation and test sets based on the validation and testing file lists provided with the data set.
- The N-TIDIGITS data set (Anumula et al., 2018), contains the spoken digits from the TIDIGITS data set, recorded on neuromorphic hardware, the spiking CochleaAMS1b sensor. In this work, only the spike trains from the single digits were used. There are two samples per for each of the 11 digits for every speaker. and the train and test sets are used as provided with the data set. They consist of 2,464 and 2,486 samples, respectively.

#### Sound processing and spike encoding

The preprocessing for the SHD data set was done similarly to previous work (Yin et al., 2021). All samples were aligned to 1 s by cutting and padding with zeros and thereafter binned in 4 ms bins to create 250 ms long samples, thus creating samples with equal lengths. The data is presented as spikes, so no further preprocessing is required. The spike trains in the N-TIDIGITS data set were downsampled and aligned to create 1,000 ms event streams for every sample.

**Figure 2:**
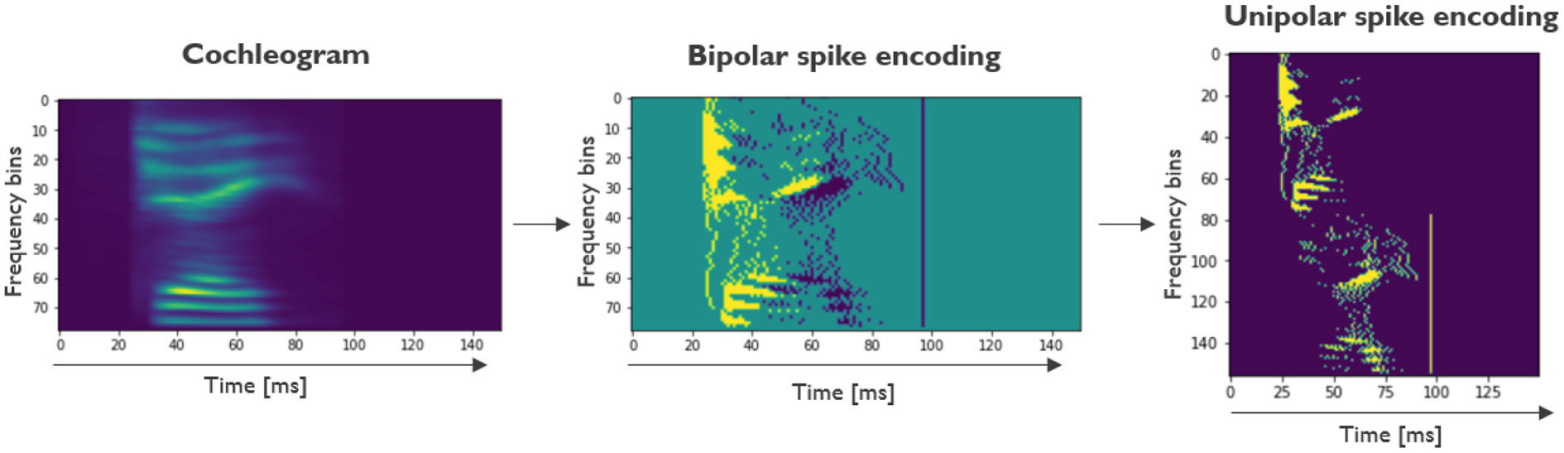
Preprocessing pipeline shown on a training sample of the TI-Alpha data set. First, the sound files are transformed into a cocheogram, where the relative intensities are shown for various frequency bins. Next, the output of the step-forward encoding is shown, a bipolar spike encoding. The last step consists of a transformation to a unipolar encoding scheme, where the positive and negative spikes are split between different channels. The unipolar encoding can directly be used as input spike trains for the LSM.

The TI-alpha and Google Speech Commands data sets are presented in .wav format. The first step in the preprocessing pipeline consists of converting the raw sound files into cochleograms. We used Librosa (McFee et al., 2015) to read out the raw sounds. As is common in LSM literature (Verstraeten et al., 2005), audio feature extraction was based on the Lyon Passive Ear model (Lyon, 1982), a model of the inner ear and the cochlea, which firstly transforms the acoustic energy into a neural representation and then acts as a non-linear filterbank. The extracted signal is then passed through a half-wave rectifier and an automatic gain module. The resulting cochleogram, containing floating-point intensities, consists of 78 channels for TI-Alpha and 86 channels for GSC. The cochleograms are normalized between 0 and 1. The subsequent step in the preprocessing pipeline is converting the cochleograms into spike trains. The spike encoding used in this work is the step-forward encoding. In this algorithm, if the next value in the sequence is above or below a baseline plus-or-minus a fixed threshold value, a positive or negative spike is registered and the baseline is adjusted to the upper and lower limit of the baseline plus-or-minus threshold. Following Petro et al. (2019), this encoding scheme is seen as the most versatile and robust among the most popular spike encoding methods and therefore our preferred method. In this work, the encoding is run separately for every frequency channel to generate the spike trains. In both the TI-Alpha and GSC data sets, the threshold parameter is empirically set to 0.005. The resulting bipolar spike encoded channels (consisting of −1, 0, or 1) are then binarized by doubling the number of channels, creating the unipolar spike encoding. The first and last half of the channels are used to represent the positive and negative spikes, respectively. In Figure 2, the full preprocessing pipeline is shown for a sample from the TI-Alpha data set.

## Results

This section discusses the results of our experiments and is organized as follows: First, we discuss the effect of increased input-inhibitory liquid neuron weights on the E/I balance and the classification performance. Next, the SFA-extended neurons are studied and, lastly, we evaluate the effect of the neuronal diversity. The Spiking Heidelberg Digits (SHD) data set is used to benchmark these results. Afterwards, the results of all extensions, called the extended LSM (ELSM), are verified on real-life speech applications (TI-Alpha and GSC data sets), and speech recorded on neuromorphic hardware (N-TIDIGITS). Lastly, the influence of the ELSM reservoir size and the computational demands are discussed for all data sets. In all experiments on all data sets the input-liquid connection probability, *p*_*in*_, was empirically set so that every liquid neuron is connected to seven input neurons. Optimizing this value was beyond the scope of this study.

### Effect of E/I balance

In this section, the effect of optimizing for the E/I balance, based on the input-liquid synaptic weight strength, is explored and compared to the LSM initialization from our reference model. Based on insights from neuroscience, E/I balance — i.e., balanced excitatory and inhibitory input currents — should lead to efficient coding and information processing. More precisely, E/I balance means that the net neuronal current, averaged over time, approaches zero. We validated this assumption for our LSM model by inspecting the per-neuron net current by means of the excitatory and the inhibitory neuron conductances for different input-inhibitory liquid neuron weight distributions. The net neuronal current is calculated by adding up the excitatory and inhibitory conductances and averaging them over time. The mean net current as well as its standard deviation are evaluated. In the reference model, the relative input-inhibitory/excitatory weight distribution ratio is equal to 1/2. In other words, the mean input-inhibitory weights are half as strong as the excitatory weights.

**Figure 3:**
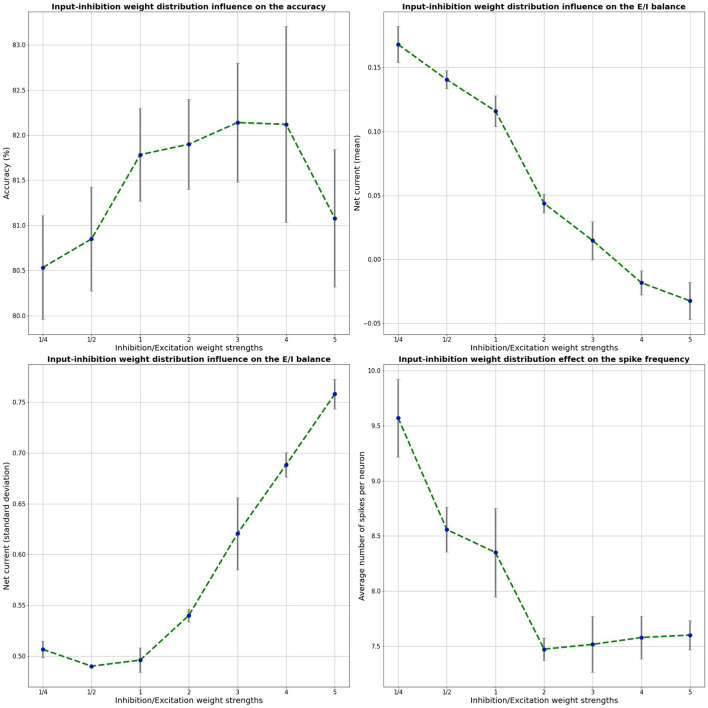
Effect of the relative input-inhibitory weight strength on the accuracy, average net current, net current standard deviation, and the number of spikes in the 2000-neuron LSM. These results were averaged over 5 trials.

Since the LSM is randomly built, our results were averaged over 5 random LSM initializations, similarly to previous work on LSM by Wijesinghe et al. (2019). The weight distributions were randomly sampled from a uniform distribution. We explore various input-inhibitory neuron weight distributions *U*[0*,factor* * 0.4], where *factor* indicates the width of the inhibitory weight distribution, relative to the excitatory weight distribution. The input-excitatory neuron weight distribution is kept to the original baseline values of *U*[0,0.4]. Both the mean of the five independent runs as well as the standard deviation are shown for the different input-liquid weight distributions in Figure 3. Here, the classification accuracy on the test set, the mean net current, the standard deviation and the number of spikes per neuron are shown for a 2000-neuron LSM on the benchmark SHD data set as a function of the input-inhibitory weights, relative to the input-excitatory weights. Naturally, since the LSM consists of random projections, we expect some variance in our results over multiple runs. It can be seen that with increasing input-inhibitory synapse weights, the E/I balance, shown by the mean net current, decreases from about 0.15–0, the point where E/I balance is found. It should also be noted that when E/I balance is in place, the accuracy on the test set reaches its optimal value. This optimum correlates with a nearly zero net mean current. This result clearly improves over the results from the baseline relative weight distribution of 1/2, which was previously used in a single-channel use-case (Maass et al., 2002). Furthermore, we note that the standard deviation further increases with the inhibitory strength, which is also found to be necessary for a deterministic chaotic dynamic regime (Van Vreeswijk and Sompolinsky, 1996) and in combination with a net zero averaged current, a predictor of edge-of-chaos dynamics in an LSM (Ivanov and Michmizos, 2021). Lastly, it is also shown that the average number of spikes per neuron is decreased by ~12% in comparison with the baseline LSM, leading to more efficient neuromorphic system. The decrease in number of spikes per neuron is stabilized for higher inhibitory-to-excitatory weight ratios due to the increase in the number of inhibitory spikes.

### Effect of spike-frequency adaptation

**Figure 4:**
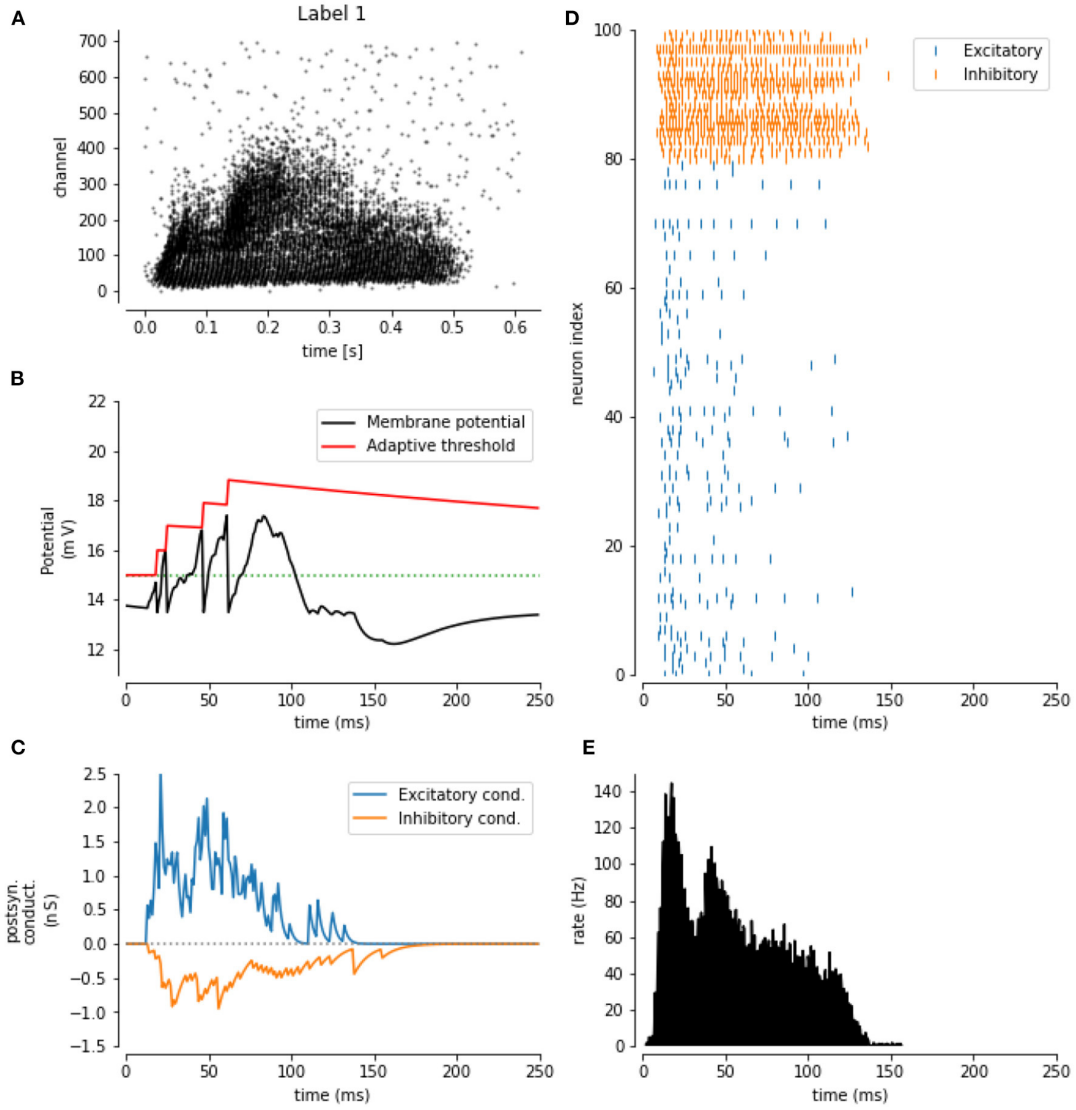
Impact of the spike-frequency adaptation: **(A)** Sample from the SHD training data set. **(B)** The evolution of the membrane potential (black) and the adaptive threshold (red) over time for one excitatory neuron. **(C)** The evolution is the excitatory (blue) and inhibitory (orange) conductance over time for the same neuron. **(D)** Output spikes for 100 sample neurons. **(E)** Momentary spike rate for the whole LSM for the SHD sample.

The next experiment consists of extending the baseline LSM containing E/I balanced input weight distributions, with the inclusion of spike-frequency adaptation (SFA). In Figure 4, the impact of SFA is shown for one sample of the SHD data set (Figure 4A). In Figures 4B,C, the response of a single excitatory neuron for this sample is shown. Here, the effect of SFA can clearly be observed around 80 ms, where no spike was generated because of the increased threshold at that time step. Furthermore, in Figure 4D, the output spikes for 100 random liquid neurons are shown on this sample, clearly establishing the sparseness of the LSM response to the input, especially for the excitatory neurons. In Figure 4E, the total amount of spikes in the LSM (all neurons) is shown as an evolution over time.

Similarly to the experiments described above, all experiments including SFA were run for five independent random LSM initializations, reducing the variance in our results. More specifically, the baseline setup (B) and an LSM without SFA but with the optimized input-liquid connections (B + E/I), are compared to an LSM equipped with SFA with a fixed time constant and with an LSM that utilizes a uniform range of SFA time constants. As described in Salaj et al. (2021), the time constant of the adaptive threshold decay, τ_*sfa*_, should be equal to the expected time where memory is required. In this case, where the expected duration of a feature in the high-dimensional projection is not known, the decay time constant is thus not evidently chosen. We experimented with three different setups: one fixed time constant of 250 *ms*, equal to the maximal duration of the input samples; the second of 550 *ms*, longer than the duration of the sample (250 *ms*); and, a uniform distribution of *U*[50,1,050] *ms*, which has a mean of 550 *ms*. The uniform distribution of time constants is aimed at both shorter and longer feature memory capacity but with the same average as the fixed case of 550 *ms*. All SFA-based experiments were performed on E/I optimized LSM designs.

The 5-run averaged effects of the inclusion of SFA on the recognition performance and the average number of spikes per neuron for the 2000-neuron LSM are shown in Table 3. Firstly, the inclusion of SFA in the E/I-optimized LSM significantly improves the classification accuracy, independent of the chosen time constant setup, over the baseline model (B) and the baseline model with E/I balanced input-liquid weight distributions (B + E/I). Secondly, there is a difference of about 1% in accuracy between one fixed decay time constant and the uniform distribution, where the bigger time constant (slower decay), outperforms the smaller one. The distribution is chosen with the aim of retaining memory for different durations. We have identified that this diversification of feature memory is useful in discriminating between different classes, leading to a more diverse liquid feature extraction than the case with a fixed time constant, and thus better results. Naturally, by increasing the spike threshold *V*_*th*_, the total number of spikes in our system decreases, making the SFA-enriched LSM even more sparse and therefore computationally efficient in comparison with the baseline. The average number of spikes does not significantly differ between the SFA setups.

**Table 3:** Effect of different SFA setups on the test accuracy and the number of spikes per neuron.

	**B**	**B + E/I**	**SFA**,	**SFA**,	**SFA**
			**small *τ_*sfa*_***	**large *τ_*sfa*_***	**(range)**
Test accuracy (%)	80.9	82.1	84.1	84.2	85.3
Spikes/neuron	8.56	7.58	5.95	5.94	6.03

B refers to the baseline model, while B + E/I refers to the baseline model with E/I balanced input-liquid weight distributions. The results for 3 different E/I optimized LSM setups with SFA are shown: small τ_*sfa*_, large τ_*sfa*_, and (range) refer to a time constant of 250, 550 ms and a uniform distribution of time constants between 50 and 1,050 ms, respectively. These results were averaged over five independent trials.

### Effect of neuronal diversity—ELSM

The last extension of the baseline LSM consists of further diversifying the liquid response by randomly sampling the membrane potential decay time constants instead of picking one fixed value. Similarly to the effect of the diversified time constants used in the SFA experiments, this leads to a more diversified liquid state response, as a neuron with low τ will be more responsive to incoming spikes, but even so will have more leak in comparison with neurons with higher τ.

In this experiment, the baseline LSM is compared to the E/I optimized LSM with and without SFA and a fixed τ of 30 *ms*, as well as compared to the same LSM with uniformly distributed membrane potential time constants: *U*[5, 55]ms, where the mean time constant is equal to the time constant in the baseline. The last version, containing all previous extensions and the diverse time constants is called the extended LSM (ELSM). The results of this experiment, averaged over five independent initializations, are shown in Table 4 for a liquid with 2000-neurons.

**Table 4:** The individual impact of all proposed extensions to the baseline LSM (B) 2000-neuron model.

	**B**	**B + E/I**	**B + E/I + SFA**	**ELSM**
Test accuracy (%)	80.9	82.1	85.3	86.1
Spikes/neuron	8.56	7.58	6.03	6.83

The final model, containing all extensions is called ELSM. These results were averaged over 5 independent trials.

First, to show the effect of the diversification of the membrane potential time constants, the optimized baseline (+ E/I + SFA) is compared to the full ELSM. There is a clear improvement in the reported classification accuracy as well as an increase in the average number of spikes per neuron in the LSM. The improvement in the reported accuracy can be related to the more diverse temporal feature set that is extracted. The diversification of the time constants was reported to have a similar effect when these time constants were learned *via* backpropagation-through-time (BPTT; Yin et al., 2021) and is related to the heterogeneity of the LSM, which was shown to improve robust learning in an SNN (Perez-Nieves et al., 2021). In our case, these time constants were however randomly picked from a uniform distribution. The increase in spike activity can be explained by the addition of “more responsive” neurons, which will have a higher probability of spiking when a presynaptic spike arrives. Finally, when comparing the baseline (Maass et al., 2002) LSM to the ELSM, it is clear that the proposed extensions result in an improvement both in terms of recognition performance, with an average increase of 5.12%, and computational efficiency, by decreasing on average 20.21% of the spikes per neuron, for a 2000-neuron LSM on the SNN benchmark SHD dataset.

### ELSM results across speech recognition data sets

To illustrate that the proposed extensions to the baseline LSM provide general enhancements, which are applicable beyond the SHD benchmark data set, all proposed extensions were validated on other speech data sets (TI-Alpha, N-TIDIGITS and GSC). As is commonly known in reservoir computing, the size of the reservoir—i.e., the size of the random high dimensional spatio-temporal projection — determines the computational power of the LSM as a whole. Scaling to larger (>2,000 neurons, our reference size) number of neurons in the liquid is an easy way to improve the results and even attain similar performances as BPTT-trained spiking neural networks, without changing any other LSM parameter. For every data set, the following procedure was followed: first, the E/I balance was achieved, then the SFA and diversification of time constants were introduced to get to the ELSM. This setup remains unchanged when the size of the ELSM is increased. The results of the baseline LSM, the ELSM, and a scaled ELSM, compared to the state-of-the-art in spiking neural networks are shown in Table 5. For the LSM implementations, the number of liquid neurons is shown in the model name.

**Table 5:** Comparison of the baseline LSM to the ELSM and the state-of-the art in spiking neural networks across different data sets.

**Model**	**Learning method**	**Accuracy (%)**
**Dataset: SHD**		
2000-LSM Baseline	Logistic regression	80.9
2000-ELSM	Logistic regression	86.1
16k-ELSM	Logistic regression	89.3
Heterog. SRNN (Perez-Nieves et al., 2021)	Surrogate gradient descent	82.7
SRNN (Yin et al., 2021)	Surrogate gradient descent	90.4
**Dataset: TI-Alpha**		
2000-ensemble LSM (Wijesinghe et al., 2019)	BP on last layer	85.0
2000-LSM baseline	Logistic regression	92.1
2000-ELSM	Logistic regression	95.0
16k-ELSM	Logistic regression	95.5
Sr-SNN (Zhang and Li, 2021)	TSSL-BP	95.6
**Dataset: N-TIDIGITS**		
512-LSM (Balafrej et al., 2022)	BP on last layer	71.3
2000-LSM baseline	Logistic regression	82.9
2000-ELSM	Logistic regression	86.3
16k-ELSM	Logistic regression	88.1
ST-RSBP (Zhang and Li, 2019b)	Spike-train BP	93.9
**Dataset: GSC (35 classes)**		
2000-LSM baseline	Logistic regression	66.7
2000-ELSM	Logistic regression	69.5
16k-ELSM	Logistic regression	83.3
64k-ELSM	Logistic regression	87.3
LSNN-SFA (Salaj et al., 2021)	E-prop	88.5

First, when comparing the baseline LSM implementation to the extended LSM (ELSM), with an equal amount of neurons, there is a clear improvement across all data sets. It should be noted that the ELSM parameterization, apart from the E/I balanced input-liquid weights, is always exactly the same, independent of the data set. The probability of input-liquid synapses, the recurrent weight distributions, the liquid topology, neuron, and synapse parameters were kept constant throughout all experiments. This shows that the proposed extensions are general improvements upon the theoretical base LSM implementation (Maass et al., 2002). Unlike our previous research on LSMs (Tsang et al., 2021), where the optimal input-liquid connection probability is derived separately for every data set, in this work an empirically set rule of thumb is used. The input-liquid connection probability is fixed in such a way that every liquid neuron is connected to ~7 randomly picked input neurons. This rule is used in all experiments and led to comparable performance to state-of-the-art models using exactly the same ELSM setup across data sets. Fine-tuning this probability was beyond the scope of this work. Only the regularization parameter (C) in the logistic regression model was updated. There is thus a clear improvement in terms of accuracy between the baseline LSM and the ELSM for different data sets. In case of the more noisy GSC data set, where the task at hand is more complex, it can see that a larger amount of neurons is required to attain similar improvements, relative to the state-of-the-art in SNNs. Since the ELSM applies a random, spatio-temporal, high-dimensional projection, a larger ELSM is required to extract all features required for better performances. This explains the smaller performance increase on the GSC data set.

Furthermore, across all data sets, scaling the liquid effectively increases the classification performance in comparison with the reference 2000-neuron ELSM. For these data sets, by increasing the liquid size, we were even able to achieve comparable performance to the state-of-the-art SNN models, trained with surrogate gradient descent. Many of these state-of-the-art models were improved with similar ideas as the ones presented in our ELSM. Spike-frequency adaptation (Salaj et al., 2021; Yin et al., 2021) and neuronal heterogeneity (Perez-Nieves et al., 2021) proved to be essential to achieve these results. In our case, this was achieved without the biologically implausible concept of backpropagation of gradients throughout the network. After network creation, the ELSM is unchanged as the random synapses are not learned. The only learning in the ELSM was performed in the output layer, where a linearly optimized logistic regression model was trained on the spike counts of the excitatory liquid neurons.

### Considerations on computational demands

In this section, we provide some initial considerations on the ELSM computational demands. The number of neurons in an ELSM is generally higher in comparison with the BPTT-learned methods in order to get to a similar classification performance. The ELSM is sparsely built, as every neuron is connected to ~9.5 other neurons on average, independent of the ELSM size, whereas in state-of-the-art BPTT-based learning methods all-to-all connected networks are used. Additionally, the ELSM spiking behavior is sparse as well, where the number of spikes per neuron per sample is significantly lower (order of magnitude less) than the reported average spike frequency BPTT SNN works (Yin et al., 2021). The combination of sparse synaptic connections and spiking activity could lead to an efficient hardware implementation on a neuromorphic chip.

Similarly to Jin and Li (2017), we investigated whether the synaptic weight resolution could be based on four bits instead of the regular 64-bit resolution, both for the input-liquid weights and the recurrent liquid-liquid synapses. The lower weight resolution would make the ELSM less computationally intensive and require less memory. We validated this concept for the 2000-neuron ELSM on all studied data sets.

**Figure 5:**
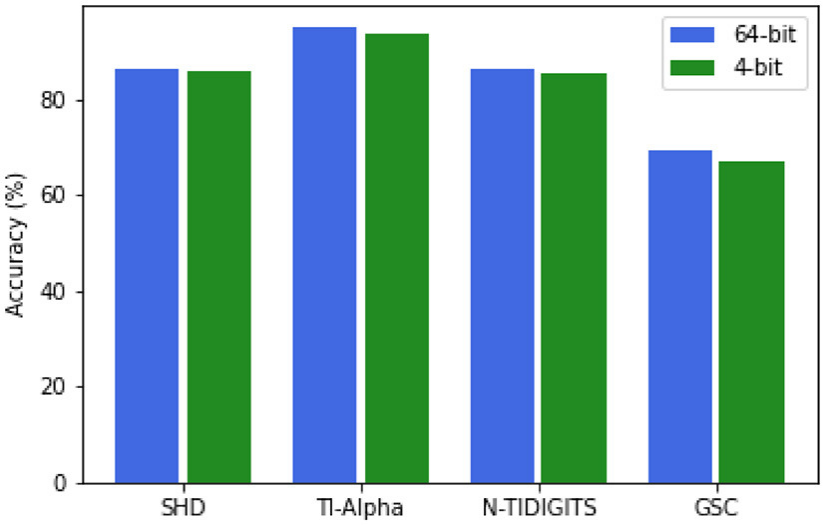
Recognition performance comparison on the test set between full 2000-neuron ELSM model (64-bit) and the lower resolution model (4-bit) across the selected data sets.

As can be seen in Figure 5, the reduction to four-bit weight resolution does not lead to a drastic drop in performance, in comparison with the original ELSM, on any of the data sets. This result can be explained by the random weight initialization. Since these weights are not trained, the smaller relative differences between them do not influence the general behavior of the liquid as much as the weights in an end-to-end trained SNNs could. The four-bit resolution suffices to differentiate between the different weight values. These results show that the ELSM weight resolution can be lowered to four-bit without significant performance losses. An extensive investigation on low-resolution weights, including a comparison with state-of-the-art SNN architectures and their implementation in hardware is a subject of future research.

## Discussion

In this work, we propose the extended LSM (ELSM) model. The ELSM brings together an optimized E/I balance leading to edge-of-chaos dynamics, improved memory capacity through spike-frequency adaptation and liquid heterogeneity. We showed that these extensions improved the computational capacity of the LSM, thereby improving the classification performance on various speech data sets while keeping the added computational cost to a minimum, even reducing the spiking activity. In contrast with other works that are focused on improving the accuracy of LSM by adapting or learning the neuronal parameters or synapses in the LSM, in this work a fixed ELSM model is proposed. After a standardized initialization procedure, the model does not require any fine-tuning or training, apart from a logistic regression classifier. This shows that the proposed ELSM model can act as a general, all-purpose, high-dimensional, spatio-temporal feature extractor. The straightforward traditional reservoir structure of and training procedure for the LSM remain unchanged, retaining the benefit of training speed and simplicity. Our experiments have thus shown that some of the brain's computational principles can be added to the basic LIF and LSM models to improve the coding efficiency and, therefore, lead to a superior performance on the selection of benchmark data sets. This shows that insights from computational neuroscience can benefit neuromorphic engineering, leading to novel research results at the crossroads of artificial intelligence and neuroscience.

Liquid state machines are seen as one of the most biologically plausible spiking neural networks but often disregarded because of their reported inferior classification performance, compared to the current state of the art in spiking neural networks—i.e., research based on backpropagation through time *via* surrogate gradients. In this work, we show that given an initialization with E/I balance, spiking LIF neurons with SFA, neuronal heterogeneity and a liquid with dimensions matching the requirements of the task at hand, the ELSM consistently outperforms the baseline LSM and even attains state-of-the-art performance on all examined speech benchmark data sets. With the ELSM, these results are achieved without the biological implausible backpropagation of gradients throughout the network. In comparison with the all-to-all connected networks with fine-tuned weights, the ELSM is larger in terms of number of neurons, yet it has a very sparse and low-precision connection matrix, as it consists of a locally connected recurrent microcircuit with sparse activations. Moreover, we illustrated that the ELSM can be effectively downsized to lower resolution without significant losses in performance.

Our study also leaves some elements that could be addressed in future research. The conductance-based leaky integrate-and-fire neuron model, introduced in this study for fair comparison with the base LSM, is more complex than the commonly used LIF neuron model. A possible next step is to verify that similar results could be attained with a simpler neuron model. Another point concerns the search for the optimal E/I balanced weight distribution. This procedure could possibly be automated by learning the synaptic weights, for instance, based on the concept of astrocytes (Ivanov and Michmizos, 2021). Additionally, topological heterogeneity, which could further diversify the liquid response (Hazan and Manevitz, 2012) and the inclusion of dendrites to the neuron model (Yang et al., 2022a), could also be a topic of investigation. Lastly, an extensive robustness study as well as an investigation on low-resolution weights and a comparison in terms of computational demands of the ELSM in relation to state-of-the-art SNNs would be of interest.

## Data availability statement

The TI-46 corpus can be purchased online. Requests to access these datasets should be directed to https://catalog.ldc.upenn.edu/LDC93S9.

## Author contributions

LD, IT, and WV contributed to development and design of the experiments. LD performed the experiments and the analysis. All authors contributed to manuscript writing, revision, read, and approved the submitted version.

## Funding

This work was supported by a grant (1S87022N) from the Research Foundation—Flanders (FWO).

## Conflict of interest

The authors declare that the research was conducted in the absence of any commercial or financial relationships that could be construed as a potential conflict of interest.

## Publisher's note

All claims expressed in this article are solely those of the authors and do not necessarily represent those of their affiliated organizations, or those of the publisher, the editors and the reviewers. Any product that may be evaluated in this article, or claim that may be made by its manufacturer, is not guaranteed or endorsed by the publisher.
